# Bias and Sensitivity Analysis When Estimating Treatment Effects from the Cox Model with Omitted Covariates

**DOI:** 10.1111/biom.12096

**Published:** 2013-11-13

**Authors:** Nan Xuan Lin, Stuart Logan, William Edward Henley

**Affiliations:** 1 Institute of Health Research, University of Exeter Medical SchoolExeter, U.K.; 2 Centre for Health and Environmental Statistics, University of PlymouthPlymouth, U.K.

**Keywords:** Bias analysis, Cox model, Omitted covariates, Sensitivity analysis, Survival analysis, Unmeasured confounding

## Abstract

**Summary:**

Omission of relevant covariates can lead to bias when estimating treatment or exposure effects from survival data in both randomized controlled trials and observational studies. This paper presents a general approach to assessing bias when covariates are omitted from the Cox model. The proposed method is applicable to both randomized and non-randomized studies. We distinguish between the effects of three possible sources of bias: omission of a balanced covariate, data censoring and unmeasured confounding. Asymptotic formulae for determining the bias are derived from the large sample properties of the maximum likelihood estimator. A simulation study is used to demonstrate the validity of the bias formulae and to characterize the influence of the different sources of bias. It is shown that the bias converges to fixed limits as the effect of the omitted covariate increases, irrespective of the degree of confounding. The bias formulae are used as the basis for developing a new method of sensitivity analysis to assess the impact of omitted covariates on estimates of treatment or exposure effects. In simulation studies, the proposed method gave unbiased treatment estimates and confidence intervals with good coverage when the true sensitivity parameters were known. We describe application of the method to a randomized controlled trial and a non-randomized study.

## 1. Introduction

Treatment or exposure effects are commonly estimated from survival or other time-to-event data using the Cox model. The gold standard design for conducting such evaluations is the randomized controlled trial because randomization acts to balance measured and unmeasured confounders. Although it is common for researchers to present unadjusted analyses, it is recommended to adjust proportional hazards models for all measured covariates in randomized studies to maximise power to detect treatment effects (Hernandez, Eijkemans, and Steyerberg, 2006). Gail, Wieand, and Piantadosi (1984) derived asymptotic formulae for the bias in estimates of treatment effects when balanced covariates are omitted from the Cox model. It was shown that when censoring is moderate, the Cox model yielded more biased estimates of treatment effect than analysis with the exponential model.

In practice, randomized experiments may be difficult to conduct for reasons of cost, logistics or ethics (Black, 1996). The increasing availability of electronic medical record databases and population-based studies is creating new opportunities for using observational data to assess the effect of medical treatments and exposures, Ghani et al., 2001;, 2009. A major challenge in using clinical databases in this way is addressing the potential bias introduced due to unmeasured differences between the treatment groups(Klungel et al., 2004). Lin, Psaty, and Kronmal (1998) presented approximate formulae for the bias due to omission of a binary or continuous confounder when estimating treatment effects from censored survival time data using the Cox model. The bias formulae were used as the basis for a method of conducting sensitivity analysis to assess how the point and interval estimates of the treatment effect vary under a range of assumptions about the unmeasured confounder. The idea behind this approach is that the plausibility of the estimated treatment effects will increase if the inferences are insensitive over a wide range of relevant scenarios.

In this paper, we develop a general framework for estimating bias and conducting sensitivity analysis when covariates are omitted from the Cox model. Formulating the problem more broadly than previous work, we consider the combined influence of three different sources of bias: (1) bias due to omitting a balanced covariate; (2) bias due to censoring; (3) bias due to the missing covariate being a confounder. The proposed approach is applicable to both randomized trials and observational studies, and provides explicit formulae for arbitrary distributions of measured and unmeasured confounders. We consider the general case in which the censoring distribution can depend on treatment or other covariates. The treatment variable can be either a binary or continuous exposure.

The paper is organized as follows. Asymptotic bias formulae, derived from the large sample properties of the partial maximum likelihood estimators, are presented in Section 2. Simulation studies conducted to investigate the accuracy of the bias formulae and to characterize the impact of the different sources of bias are presented in Section 3. Section 4 discusses how the bias formulae can be used to develop a new method of sensitivity analysis for treatment effects in proportional hazards models. The method is applied to data from a randomized controlled trial and a non-randomized study in Section 5.

## 2. Bias Formulae

We denote random variables by upper case letters and their values by lower case letters. Suppose 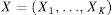 are *K* measured covariates with joint distribution 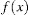, and 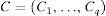 are *q* unmeasured covariates with conditional joint distribution 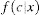. Let *T* and 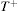 represent the true event/failure time and possible censoring time respectively. We assume failure and censoring times are independent conditional on *x* (i.e., 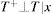). We observe 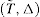, where 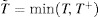, and 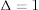 if 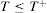 and 0 otherwise. The true hazard is assumed to be
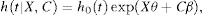
1where 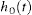 is the baseline hazard function and 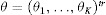 and 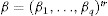 are coefficients for *X* and *C*, respectively. But since *C* is omitted, one is forced to fit the model
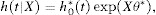
2where 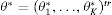 are the coefficients when *C* is missing. Let 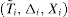 be 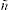 independent replicates of 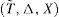. Then the average partial log-likelihood based on (2) is
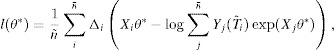
3where 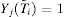 if 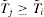 and 0 otherwise. It is shown in Web Appendix A that as 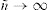, the score function 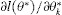 has the limit
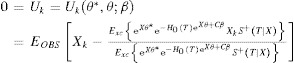
4for 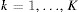, where 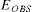 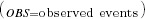 is the mean over the uncensored subjects, 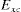 is under the density 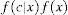 and 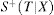 is the survival function of censoring time conditional on *X*. Inclusion of 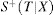 allows the censoring distribution to depend on covariates.

The system of equations 1988, 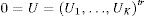, relate 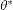 and 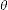, and therefore the asymptotic biases 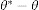 can be evaluated from them. The first-order Taylor series approximation is
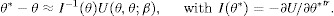
5

### 2.1. The Distributions of Uncensored Subjects

Let
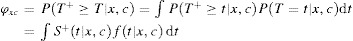
be the uncensoring probability conditional on *x* and *c*, where 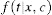 is the density of model (1) and 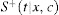 is the survival function of censoring time.

The density of the observed event times is then given by
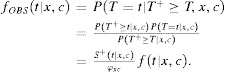


The mean of 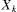 for uncensored subjects is
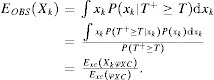
6

### 2.2. Extension of the Results of Lin et al. (1998)

Lin et al. (1998) proposed bias formulae for survival analysis with unmeasured confounders based on the assumption of rare events (small 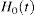) or small 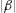. For binary *x*, the proposed bias approximation is
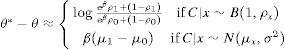
7The simulation of Lin et al. (1998) showed that 2002 are good approximations when 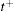 was generated from the uniform 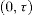 distribution and the censoring percentage is 90%.

Using the assumption of rare events and the simulation settings in Lin et al. (1998), Web Appendix B shows that Equation 1988 reduces to a simple equation of 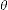 and 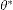:
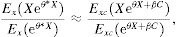
8which leads to the formulae 2002 when 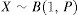 and 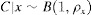 or 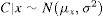. The Equation 2006 therefore provides a general extension of the results in Lin et al. (1998) to arbitrary distributions of *X* and *C*.

## 3. Bias Analysis

### 3.1. Bias Analysis for a Binary Treatment with a Single Omitted Covariate

We now show the asymptotic formula for the bias in the important special case of a single missing covariate *C* and a binary exposure variable *X* taking values 1 or 0 with probabilities *p* and 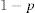, respectively.

The Equation 1988 leads to (see proof in Web Appendix C)
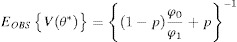
9with
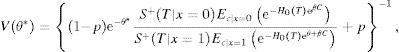
where the expectations 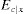 and 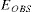 are under 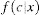 and
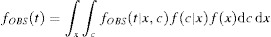
respectively, and 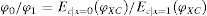 is the ratio of uncensoring rates between control and treatment groups.

From 2004, it can be seen that the relation between 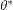 and 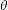 mainly depends on three factors (corresponding to the three sources of bias): the effect of the missing covariate, 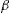; censoring mechanism, 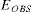, 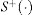 and 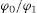; and the ratio of conditional expectations, 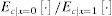. The latter ratio represents how much the density of *C* varies between 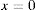 and 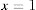 and, hence, measures the extent to which *C* is a confounder.

The bias is also affected by the cumulative baseline hazard function 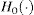. But if times are not censored, 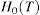 is an exponential variable with the rate 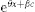 and 2004 reduces to
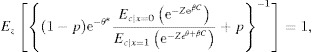
10where 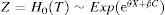. As a result, the bias is independent of the form of 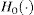 in the absence of censorship.

When 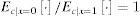, *C* is not a confounder. In this case, Equation 1991 shows that 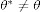 and, consequently, the MLE of the Cox model is still biased even if *C* is a balanced covariate. Bretagnolle and Huber-Carol (1985) studied the bias in this case and showed that the estimated effect is biased toward zero as 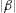 increases. This is because the event times with 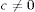 tend to zero as 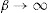 and tend to 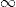 as 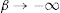. Consequently the subjects with 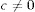 cannot provide information about 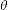 in the limiting case. However, the subjects with 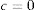 do still supply information about 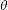 and hence the limit of 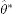 as 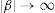 is not zero for binary *C*. An illustration of this explanation is given in Web Figure 1.

**Figure 1:**
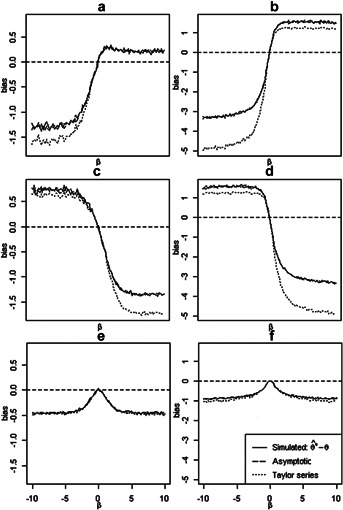
Comparison of simulated biases, asymptotic biases and first-order Taylor series approximations for different types of omitted covariate and censorship. Since 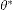 is the asymptotic value of the MLE 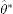 and the sample size=10,000 is large, we calculated the simulated bias by 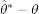. The asymptotic biases and Taylor series approximations were obtained from 2004 and 2001, respectively. Monte Carlo integration was used to approximate the expectations in formulae. (a) Binary confounder *c*: (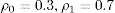), censored; (b) Normal confounder *c*: (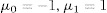), censored; (c) Binary confounder *c*: (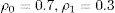), censored; (d) Normal confounder *c*: (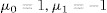), censored; (e) Binary balanced *c*: (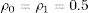), uncensored; (f) Normal balanced *c*: (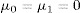), uncensored.

Following 2008, the first-order Taylor series approximation is
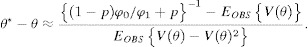
11

### 3.2. Accuracy of Asymptotic Formulae and Taylor Series Approximations

Figure 1 shows a comparison of the asymptotic and simulated biases and Taylor series approximation under the influence of different sources of bias. We generated 10,000 *x* from 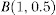. The confounder *C* was generated from 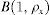 for the binary confounder, and from 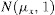 for the normal confounder. The event times *t* were generated from model (1) with 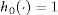, 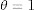 and 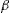 taking 100 sequence values from 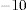 to 10. For the censoring cases, we let 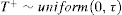 with 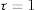. The observed times were given by 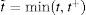.

Figure 1 shows that the simulated and asymptotic biases are seen to agree closely, confirming that these asymptotic formulas adequately describe the biases. The accuracies of the Taylor series approximations decrease as 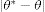 gets large, because the approximation error is of the order 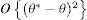.

For more modest values of 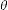, for example 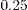 and 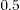, the biases will have similar patterns but be shifted up as 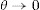 (see Web Figures 2 and 3). In Web Figure 8, we let 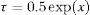 and 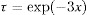 to allow the distribution of censoring to depend on treatment group. The figure illustrates how different choices of censoring function can impact on the biases.

### 3.3. Bias of Omitting a Balanced Covariate in Randomized Studies

Figure 1e and f show the biases when a balanced covariate is omitted. It is clear that omission of a relevant covariate leads to biased treatment estimates for the Cox model, even in randomized studies.

The reason is that the parameters 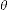 and 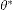 are measuring different features of the population. When we model the hazard as
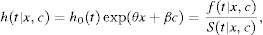
the interpretation of 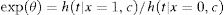 is the hazard ratio between 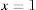 and 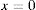 while the values of *c* are fixed. But in randomized studies (where we assume 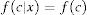), when we model the marginal hazard as
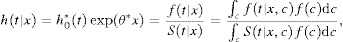
12the interpretation of 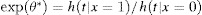 is the hazard ratio between 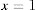 and 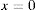 while *c* is marginalized. Similarly, 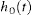 is the hazard when 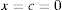, and 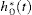 is the hazard when 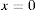 and *c* is marginalized. The superscript 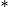 emphasizes that they do not have the same interpretation.

When *c* is integrated out, the marginal hazards 2008 for 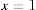 and 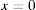 are not proportional over time, and the MLE of 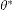 represents an average over time of the log marginal hazard ratios between 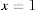 and 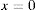 (Lin and Wei, 1989). Therefore, it will lead to bias if we use a marginal hazard ratio 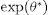 to estimate a hazard ratio 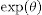. In randomized studies, as outlined in Section 2001, usually 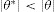 and 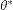 will attenuate to some limit between 0 and 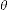 as 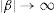.

### 3.4. The Limits of Biases as 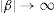


One phenomenon that can be noticed from Figure 1 is that all biases increase with 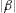 but always tend to some limits, no matter if *C* is a confounder or not. The reason is that the marginal hazard ratio has finite limits as 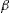 tends to 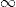 and 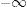. For example, for 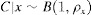, the marginal hazard is
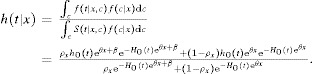
The ratio, 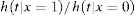, tends to 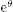 as 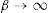 and
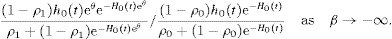


### 3.5. The Effect of Censoring

**Figure 2:**
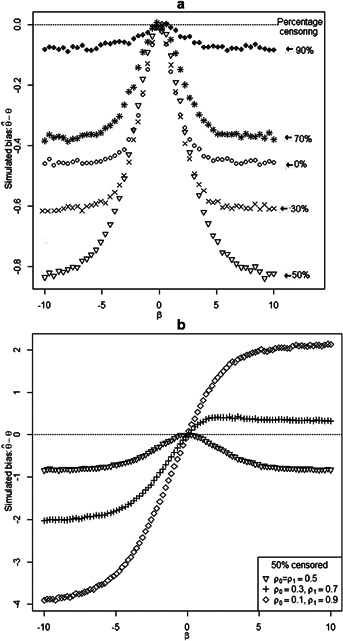
The effect of overall censoring and confounding on bias: (a) biases of omitting a balanced covariate where 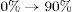 data are censored; (b) biases under different strengths of confounding, 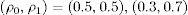 and 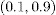 when 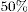 data are censored.

Figure 2a shows the effect of censoring on the bias of omitting a balanced covariate. The event times were generated from (1) with 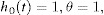 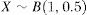 and 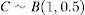. The possible censoring times 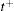 were simulated from uniform 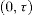 with 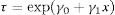.

Following the result A-4 in Web Appendix, the uncensoring probability can be written as
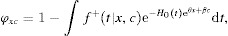
where 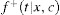 is the density of possible censoring times.

Under the simulation settings, 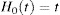 and 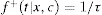. The probability of censoring conditional on *x* is thus
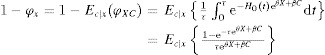
13The values of 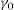 and 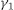 were then solved from [Bibr b13] such that the probabilities of censoring were the same for 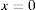 and 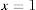 and could be 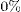, 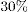, 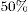, 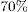, and 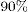. The number of event times *n* was fixed at 100,000 and the total sample size was 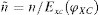.

Figure 2a shows that censoring influences the bias in two different ways. The bias increases as the censoring percentage increases from 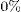 to 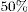, but decreases as the censoring percentage increases from 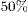 to 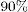. The bias is plotted for a wider range of censoring percentages in Web Figure 4.

The reason for this inconsistent effect of censoring is as follows: when the censoring percentage increases (0–50%) and 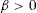, the subjects with 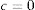, which provide most of the information about 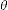, are likely to be censored, and consequently, the bias is increased. But as the censorship rate increases further (50–90%), almost all of the few events occur with 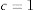 and almost all the times with 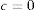 are censored. So nearly all the subjects supplying information about 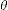 have the same value of 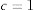 (Chastang, Byar, and Piantadosi, 1988). If the sample size is sufficiently large, the bias will tend to zero as the censoring percentage tends to 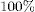. A similar explanation applies for 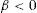. An illustration of this explanation is given in Web Figure 5.

### 3.6. The Effect of Confounding

Of particular relevance to non-randomized studies, we considered the influence of different levels of confounding on the bias function when 50% of the data are censored (Fig. 2b). We generated 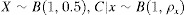 and consider three scenarios with 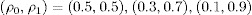. The difference 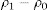 represents the imbalance of the distributions of 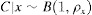 between 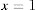 and 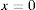 and so measures the strength of confounding. As 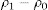 increases, it can be seen that the estimate is biased upwards for 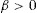 and downwards when 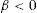. For the case 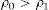, the bias would be affected in the other direction.

### 3.7. The Effect of Additional Measured Covariates

In practice, the analyst is likely to have access to additional measured covariates (possibly confounders) that would need to be adjusted for, in addition to the exposure variable *X* (and the unmeasured confounder, *C*).

**Figure 3:**
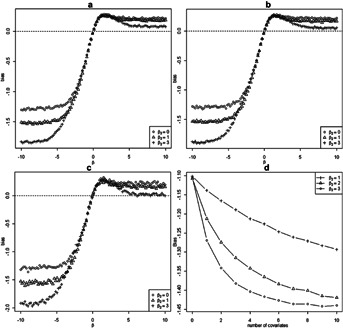
The effect of additional measured covariates on the simulated bias 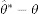: (a) 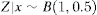; (b) 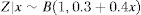; (c) 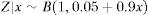; (d) the effect of increasing the number of measured covariates on the simulated bias when 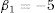 and 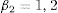 and 3.

Under the approach of Lin et al. (1998), an additional covariate *Z* does not affect the bias if the mean of *C* conditional on *x* and *z* is additive in *x* and *z*, that is 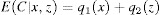 (VanderWeele, 2008). However, our simulation results in Figure 3a–c show that an additional covariate may introduce a small degree of bias when 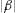 is large. We generated 100,000 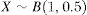 and 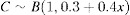. The additional covariate *Z* was simulated from 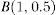, 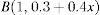 and 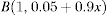 for Figure 3a–c, respectively. Under these data-generating processes, 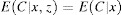 and the additivity assumption is satisfied.

A sample of 100,000 survival times was generated from 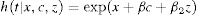 and 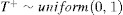. The data were then fitted by the reduced model 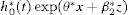. It can be seen that the bias is not impacted by the distribution of *Z*, but is affected by 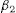, when 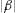 is large. The results were similar when we allowed censoring to depend on *X* and *Z* by assuming 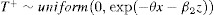 (see Web Figure 9).

It is then natural to investigate the influence of more than one additional covariate when 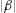 is large. To simplify the problem, we examine the case where all the additional covariates are binary and independent of each other, with the same coefficient 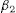. As the bias is only significant for large negative 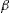, we set 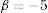. The results displayed in Figure 3d, show the bias increases slightly with the number of covariates and the increments are not linear.

## 4. Sensitivity Analysis

The aim of our proposed method of sensitivity analysis is to assess how the point and interval estimators for 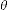 or associated *P*-value would change given clinically plausible values of the sensitivity parameters 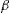 and 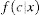.

### 4.1. Point Estimate

For a sample with 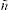 observed times 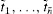, of which *n* are uncensored 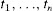, from 1988 we have the relation between 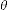 and 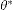 approximately relies on the equations 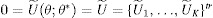 with
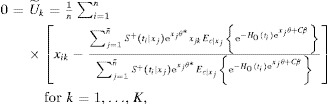
14where the expectation 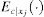 can be calculated analytically or approximately with respect to 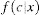.

Write 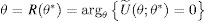. Due to the functional invariance property of MLE, the point estimate of the true value 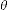 is then 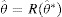. The function 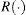 and its inverse 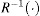 relate 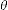 and 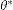, and play a key role in sensitivity analysis.

The baseline survivor function 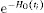 in [Bibr b14] is estimated by solving
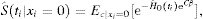
where 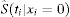 is the Breslow (1972) estimator:
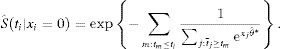
The survival function of censoring can be also approximated by the Breslow (1972) estimator by considering events as “censored” observations and censored observations as “events” (Satten and Datta, 2001).

### 4.2. *P*-Values

In many applications, we are interested in evaluating the evidence the data give about a null hypothesis 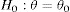 (for example, that a hazard ratio equals one). Using 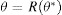, this null hypothesis is equivalent to 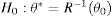 and the two-sided *P*-value is therefore
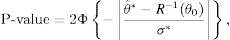
15where 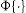 is the cumulative distribution function of 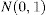 and 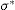 is the standard error of 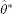.

### 4.3. Confidence Intervals

Since the distribution of 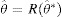 might be slightly skewed (see example in Web Figure 6), the traditional way of using standard error to calculate confidence intervals (CI) could be misleading. An alternative way is to construct CI by the highest density interval. To do this, we generate *B* bootstrap samples 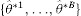 from the multivariate normal distribution 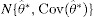, where 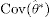 is the covariance matrix of 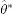. The sample of the *k*th parameter 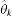, 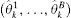 is then obtained from 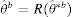 for 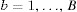. The highest density interval of 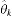 can be computed from the sample 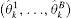 by using the emp.hpd function in the R package TeachingDemos.

**Table 1:** Simulated bias of point estimates and coverage of 95% confidence intervals for the hazard ratio associated with treatment under two methods of sensitivity analysis, when censoring is moderate. The equations [Bibr b14] and [Bibr b16] were used to estimate 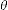 and its confidence interval in the last two columns

						unadjusted		Lin et al. (1998)		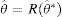	
								
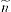	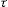	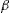	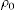	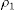	Fraction Censored (%)	Bias	Coverage (%)	Bias	Coverage (%)	Bias	Coverage (%)
100	0.56	1	0.1	0.9	50	0.78	39	0.01	97	−0.04	95
	0.57		0.3	0.7	51	0.35	83	−0.02	91	0.00	98
	0.58		0.5	0.5	51	−0.08	90	−0.08	90	−0.03	99
	0.35	2	0.1	0.9	50	1.38	3	−0.04	96	0.07	96
	0.36		0.3	0.7	50	0.42	78	−0.21	87	−0.06	97
	0.35		0.5	0.5	50	−0.24	82	−0.24	82	0.01	100
	0.20	3	0.1	0.9	50	1.71	0	−0.12	91	0.15	91
	0.22		0.3	0.7	49	0.36	84	−0.40	72	−0.11	99
	0.21		0.5	0.5	50	−0.44	68	−0.44	68	0.00	100
500	0.57	1	0.1	0.9	50	0.76	0	−0.02	95	0.00	95
	0.58		0.3	0.7	50	0.32	42	−0.06	90	−0.01	98
	0.57		0.5	0.5	50	−0.10	90	−0.10	90	−0.01	99
	0.34	2	0.1	0.9	50	1.27	0	−0.15	82	−0.04	92
	0.34		0.3	0.7	50	0.43	11	−0.20	70	0.03	99
	0.34		0.5	0.5	50	−0.30	44	−0.30	44	0.01	100
	0.20	3	0.1	0.9	50	1.65	0	−0.18	81	0.04	89
	0.20		0.3	0.7	50	0.38	21	−0.38	28	−0.02	100
	0.21		0.5	0.5	50	−0.48	4	−0.48	4	0.01	100
1000	0.57	1	0.1	0.9	50	0.73	0	−0.04	93	−0.02	96
	0.57		0.3	0.7	50	0.30	11	−0.07	89	0.01	99
	0.58		0.5	0.5	50	−0.10	80	−0.10	80	−0.01	100
	0.34	2	0.1	0.9	50	1.29	0	−0.13	80	0.00	94
	0.34		0.3	0.7	50	0.40	1	−0.23	41	0.00	99
	0.34		0.5	0.5	50	−0.30	7	−0.30	7	−0.01	100
	0.20	3	0.1	0.9	50	1.65	0	−0.18	65	0.03	90
	0.20		0.3	0.7	50	0.40	0	−0.36	6	0.00	99
	0.21		0.5	0.5	50	−0.49	0	−0.49	0	−0.01	100

However, the bootstrap method may become computationally inefficient, when the dimension of 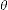 is high (e.g., 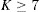). We thus give an approximation by using the confidence bounds of 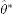. Suppose we are interested in the parameter 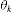 and its confidence interval 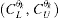. As shown in Section 1989, the effect of additional measured covariates is negligible. It means that the solution of 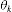 would not change appreciably if we ignore all the covariates except 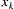 in [Bibr b14].

In addition, 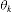 is usually a monotonically increasing function of 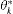 in practice. Let 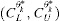 be the confidence interval of 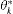. The lower bound 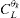 then can be estimated from the equation
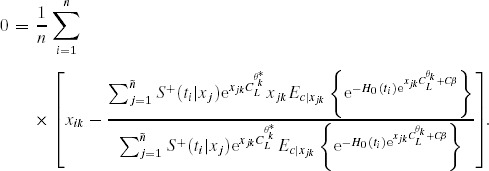
16Similarly, 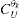 can be obtained from the above equation by substituting 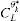 by 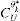. Our simulation shows that this approximation is sufficiently accurate and very efficient.

### 4.4. Simulation Study

Lin et al. (1998) proposed a simple method for sensitivity analysis. Here we conducted simulation studies to compare their method with our approach.

Table 1996 shows the biases of point estimators and coverage of 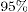 CIs in 1,000 simulation replications, when given the true 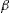 and 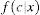. To compare with the method of Lin et al. (1998), we used similar simulation settings to theirs: 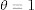, 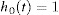, 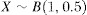, 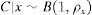 and 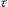 was solved from [Bibr b13] so as to ensure moderate levels of censorship (fraction censored was about 50%). It is clear that our proposed method gives almost unbiased point estimates and good coverage of confidence intervals. The method of Lin et al. (1998) gets worse as 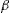 increases, because it only addresses the bias attributable to confounding. The results for light (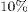) and heavy (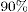) censorships are presented in Web Tables 1 and 2, respectively. We note that both methods of sensitivity analysis gave biased treatment estimates when censoring was heavy and the sample size was small (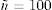). However, since the accuracy of approximation [Bibr b14] increases with the number of observed events, the proposed method is asymptotically unbiased irrespective of the censoring rate. The minimum sample size at which the method achieves approximately unbiased estimates increases with the censoring rate, and for a censoring rate as high as 90% is about 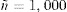.

## 5. Real Examples for Sensitivity Analysis

### 5.1. Vitamin and Minerals Trial

Ellis et al. (2008) conducted a randomized controlled trial assessing the effect of antioxidant and folinic acid supplementation on developmental outcomes for children with Down syndrome. Comparing infants allocated to folinic acid (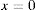) with those who were not (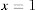), the estimated hazard ratio for age of sitting was 1.25 (95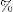 confidence interval 0.88–1.78). These results did not change appreciably after adjustment for area of residence, maternal ethnicity, birth weight, and social class.

We now assess the impact on the treatment estimates for age at sitting of assuming a binary confounder, *c*, has been omitted from the model, where 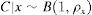. As this is a randomized controlled trial and any random imbalance in the prevalence of the unmeasured confounder between treatment groups is likely to be small, we restrict 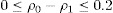. Assuming the true prevalence of the omitted covariate for treatment groups combined is 0.5, the probability of a confounding effect 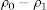 of more than 0.2 by chance is 0.02 for the trial sample of size 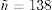.

**Figure 4:**
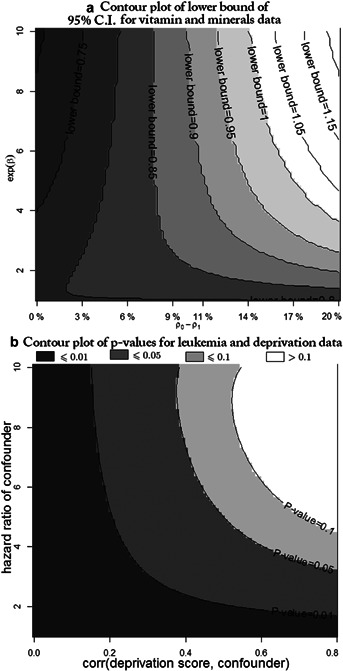
Contour plots of sensitivity analysis results: (a) the lower bounds of the 95% confidence intervals (use [Bibr b16]) for the hazard ratio of folinic acid on age of sitting for children with Down syndrome; (b)the *P*-values (use (15)) for the two-sided test that the log-hazard ratio of deprivation score 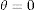.

Figure 4a shows the sensitivity of the lower limit of the confidence interval for the hazard ratio of folinic acid to adjustment for an unmeasured binary covariate of specified properties, where we set 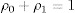. For 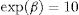, the difference in probabilities 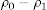 must be 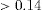 for the treatment effect to become significant. The same conclusion can be obtained from the contour plot in Web Figure 3 which shows results of a similar sensitivity analysis for the *P*-value of the treatment estimate. The results for antioxidant supplementation in Web Table 3 show that the treatment effect is significant only when 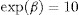 and 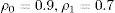. Given the nature of the study design, the conditions required for the treatment effects to be significant are implausible, suggesting that the original findings of non-significance are robust to the presence of realistic levels of unmeasured confounding.

A simulation study was conducted with similar sample size and censoring rates to the vitamin and mineral trial, providing support for the validity of the treatment estimates presented in the sensitivity analysis (see Web Table 4). However, we note that in this illustrative application, the width of the confidence intervals suggests the sensitivity analysis, in common with the original analysis, lacks power to establish statistical significance for small studies.

### 5.2. Leukaemia and Deprivation Study (Non-Randomized)

Henderson, Shimakura, and Gorst (2002) analyzed the effect of a social deprivation score *X* (where lower values indicate less affluent areas) on the time in years since diagnosis with acute myeloid leukemia to death (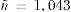). The estimated hazard ratio for a 1 point increase in *x* was 1.03 (*P*-value = 0.0012) after adjustment for age, gender and white blood cell count, indicating that prognosis is less good if the patient lives in a more deprived residential location.

We now consider a potential unmeasured binary confounder *C*, which affects both survival time *T* and the deprivation score *X*. We generated *c* from 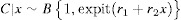, where 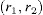 were solved from
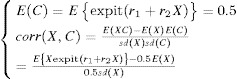
such that the marginal distribution is 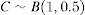 and the desired 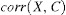 is obtained.

Figure 4b shows the sensitivity of *P*-value for different choice of 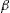 and 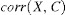. It shows that even if the correlation is strong, that is 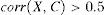, the hazard ratio of the confounder needs to be 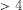 for the hazard ratio of *x* to become non-significant at the 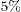 level. It seems unlikely that such an important covariate would be missed, suggesting that the original finding of a significant effect of deprivation score is robust to the presence of realistic levels of unmeasured confounding.

A simulation study was conducted with the same sample size (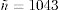), covariate *X* and censoring rate (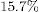) as this non-randomized study. To extend the range of scenarios considered, survival times were simulated assuming the true value of 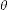 was 0 (i.e., assuming the continuous exposure has no effect on survival). Here the emphasis was on comparing the extent to which the sensitivity analysis methods avoid false rejection of the null hypothesis 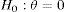. The results are summarized in Web Table 5 and provide further support for the validity of the proposed formulae when applied to data from non-randomized designs.

## 6. Discussion

**Figure 5:**
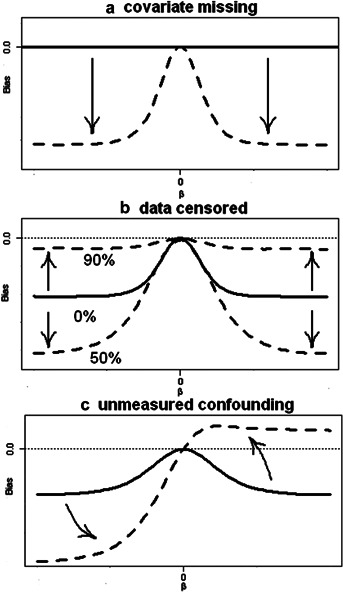
An illustration of the influence of the different sources of bias when estimating binary treatment effects from the Cox proportional hazards model with an omitted binary covariate. (a) solid: no missing data, no bias; dashed: bias due to omitting a balanced covariate. (b) solid: bias due to omitting a balanced covariate; dashed: bias due to omitting a balanced covariate and censoring. (c) solid: bias due to omitting a balanced covariate and censoring; dashed: bias due to omitting a confounder and censoring.

We explored a general framework for assessing bias in treatment estimates from the Cox model with omitted covariates. Bias formulae based on asymptotic properties of the likelihood estimator were presented and validated in simulation experiments. The results showed that the confounding biases for censored survival data are typically complicated. However, the proposed approach made it possible to describe the influence of three different sources of bias: omission of a balanced covariate, data censoring and unmeasured confounding. Figure 5 characterises the sources of bias:

1. In thei absence of a missing covariate, the bias curve remains at zero (the solid line); when a balanced covariate is omitted, the effect is underestimated to a limit as 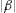 increases (the dashed line).
2. When the data are censored, the bias is maximized at 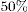 censoring but decreases with heavy censorship.
3. When the missing covariate is a confounder, the shape of bias changes. If the association between *x* and *c* is positive, the limits increase on the right side but decrease on the left side, and hence the slope of bias increases. Conversely, if the association between *x* and *c* is negative, the limits decrease on the right side but increase on the left side.

Although the bias formula is applicable under a range of assumptions, this paper has focused on considering the simple case of a binary exposure and a single unmeasured confounder. Further simulation work showed that the bias increased slightly in the presence of one or more measured confounders for large values of 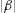. The extension to multiple unmeasured confounders is straightforward. If there are several missing covariates 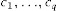 with coefficients 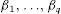, then we can interpret *c* as the composite score, 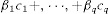, with 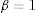 (Lin et al., 1998). Lin et al. (1998) also argue that the choice of a single unmeasured confounder is a less severe restriction when all the known confounders are adjusted for in the survival model.

The bias formula was used as the basis for proposing a new method to assess the sensitivity of estimates of treatment effects to omission of relevant covariates. Simulation experiments were conducted to compare the method with the approach of Lin et al. (1998), a special case of the proposed method when the rate of censoring is high. The method of Lin et al. (1998) has the benefit of ease of implementation, being based on a simple adjustment formula, but its relative performance deteriorates as the magnitude of 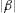 increases. In contrast, the simulations indicate that the proposed method can provide sufficiently unbiased treatment estimates, and associated confidence intervals with good coverage, over a wide range of scenarios, when the true sensitivity parameters 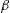 and 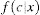 are known.

Sensitivity analysis is a flexible approach to addressing omission of covariates that makes it possible to assess the impact of ’clinically plausible’ levels of unmeasured confounding and other sources of bias on the treatment estimates (Groenwold, 2010). However, it does not provide a single precise estimate of treatment effectiveness nor does it help identify the nature of any bias from omitting covariates. A number of alternative strategies for tackling unmeasured confounding have been proposed that do attempt to provide explicit estimates of causal effects. An overview of these different methods was given in Aleyamehu et al (1996), including instrumental variables and the prior event rate ratio method (Tannen et al., 2009).

The method of sensitivity analysis proposed in this paper could be extended in a number of ways. First, incorporating adjustment for the propensity score into the sensitivity analysis would provide an efficient way of controlling for the effect of measured covariates (Rosenbaum, 1991). Other possible developments include consideration of specific distributional forms (both univariate and multivariate) for the unmeasured confounder(s) to provide special cases of the generic bias formulae for a wider range of common confounding models.

Omission of relevant covariates is a common source of bias when estimating treatment or exposure effects from survival data. Although we cannot directly adjust for unmeasured covariates, their potential impact can be assessed by means of sensitivity analyses. Indeed, Groenwold et al. (2010) argue that all analyses of causal associations in observational data should include an assessment of robustness to unmeasured confounding. The current study provides new tools for conducting sensitivity analysis for survival outcomes, with applicability to both randomized controlled trials and observational studies. Implementation of the methods requires numerical evaluation of the appropriate bias formulae. This can be achieved using Monte Carlo methods and illustrative R code is available on request from the authors.

## 7. Supplementary Materials

Web appendices, tables and figures referenced in Sections 2, 2.2, 3.1, 3.2, 3.5, 4.3, 4.4, 5.1 and 5.2 are available with this paper at the Biometrics website on Wiley Online Library.

## Supporting Information

All Supplemental Data may be found in the online version of this article.
