# Supplementary material for: Bias and Sensitivity Analysis When Estimating Treatment Effects from the Cox Model with Omitted Covariates
Source: Biometrics. 2013 Nov 13;69(4):850–60. doi: 10.1111/biom.12096 (PMC4230475; doi:10.1111/biom.12096)
Supplement: Supplementary file 1 — Supporting Information. [file biom0069-0850-sd1.pdf]

Web-based Supplementary Materials for Bias and Sensitivity Analysis when Estimating Treatment Effects from the Cox Model with Omitted Covariates

Web Appendix A: Proof for (4)

The kth component of the score function can be written as the sequence

$$U_{\tilde{n}k}(\theta^*) = \frac{1}{\tilde{n}} \sum_{i=1}^{\tilde{n}} \Delta_i \left(X_{ik} - \frac{A_{\tilde{n}ik}}{B_{\tilde{n}i}} \right) \quad \text{for} \quad \tilde{n} = 1, 2, ...; k = 1, ..., K,$$

where

$$A_{\tilde{n}ik} = \frac{1}{\tilde{n}} \sum_{j=1}^{\tilde{n}} Y_j(\tilde{T}_i) e^{X_j \theta^*} X_{jk} \quad \text{and} \quad B_{\tilde{n}i} = \frac{1}{\tilde{n}} \sum_{j=1}^{\tilde{n}} Y_j(\tilde{T}_i) e^{X_j \theta^*}.$$

Although the model (2) is incorrect, the MLE $\hat{\theta}^*$ converges to a well-defined constant (Lin and Wei, 1989). For simplicity of notation, we use the same θ^* to represent this limit.

$A_{\tilde{n}ik}$ and $B_{\tilde{n}i}$ are sums of i.i.d random variables with conditional expectations

$$A_{ik} = P(\tilde{T} \geq \tilde{T}_i) E_x \left(e^{X\theta^*} X_k | \tilde{T} \geq \tilde{T}_i \right)$$

and

$$B_i = P(\tilde{T} \geq \tilde{T}_i) E_x \left(e^{X\theta^*} | \tilde{T} \geq \tilde{T}_i \right)$$

respectively.

By the weak law of large numbers, $A_{\tilde{n}ik}$ and $B_{\tilde{n}i}$ converge in probability to A_{ik} and B_i respectively as $\tilde{n}$ tends to infinity.

Using theorems (2.2), (2.4) and (2.6) in Jiang (2010, page 22-23), the ratio $A_{\tilde{n}ik}/B_{\tilde{n}i}$ converges in probability to

$$D_{ik} = \frac{A_{ik}}{B_i} = \frac{E_x\left(e^{X\theta^*}X_k|\tilde{T} \geq \tilde{T}_i\right)}{E_x\left(e^{X\theta^*}|\tilde{T} \geq \tilde{T}_i\right)}$$
$$= \frac{\int_x e^{x\theta^*}x_k P(x|\tilde{T} \geq \tilde{T}_i)dx}{\int_x e^{x\theta^*}P(x|\tilde{T} \geq \tilde{T}_i)dx},$$

where

$$P(x|\tilde{T} \geq \tilde{T}_i) = \frac{P(\tilde{T} \geq \tilde{T}_i|x)P(x)}{P(\tilde{T} \geq \tilde{T}_i)} = \frac{P(\min(T, T^+) \geq \tilde{T}_i|x)P(x)}{P(\tilde{T} \geq \tilde{T}_i)}.$$

Under the assumption $T \perp T^+|X$,

$$P(x|\tilde{T} \geq \tilde{T}_i) = \frac{P(T \geq \tilde{T}_i|x)P(T^+ \geq \tilde{T}_i|x)f(x)}{P(\tilde{T} \geq \tilde{T}_i)} = S(\tilde{T}_i|x)f(x)\frac{S^+(\tilde{T}_i|x)}{P(\tilde{T} \geq \tilde{T}_i)},$$

where

$$S(\tilde{T}_i|x) = E_{c|x}\left\{S(\tilde{T}_i|x, C)\right\} = \int_c e^{-H_0(\tilde{T}_i)e^{x\theta+c\beta}}f(c|x)dc$$

is the survival function conditional on x, $H_0(\cdot)$ is the cumulative baseline hazard, and $S^+(\tilde{T}_i|x)$ is the survival function of censoring time given x.

It follows that

$$D_{ik} = \frac{\int_x e^{x\theta^*}x_k S(\tilde{T}_i|x)f(x)\frac{S^+(\tilde{T}_i|x)}{P(\tilde{T}\geq\tilde{T}_i)}dx}{\int_x e^{x\theta^*}S(\tilde{T}_i|x)f(x)\frac{S^+(\tilde{T}_i|x)}{P(\tilde{T}\geq\tilde{T}_i)}dx}$$
$$= \frac{\int_x\int_c e^{x\theta^*}x_k e^{-H_0(\tilde{T}_i)e^{x\theta+c\beta}}S^+(\tilde{T}_i|x)f(c|x)f(x)dxdc}{\int_x\int_c e^{x\theta^*}e^{-H_0(\tilde{T}_i)e^{x\theta+c\beta}}S^+(\tilde{T}_i|x)f(c|x)f(x)dxdc}$$
$$= \frac{E_{xc}\left\{e^{X\theta^*}X_k e^{-H_0(\tilde{T}_i)e^{X\theta+C\beta}}S^+(\tilde{T}_i|X)\right\}}{E_{xc}\left\{e^{X\theta^*}e^{-H_0(\tilde{T}_i)e^{X\theta+C\beta}}S^+(\tilde{T}_i|X)\right\}}.$$

In the special case when $S^+(\tilde{T}_i|X) = 1 - \tilde{T}_i/\tau_X$ for $0 \leq \tilde{T}_i \leq \tau_X$, D_{ik} will be an indeterminate form 0/0 as $\tilde{T}_i$ approaches τ_X. In this case, l'Hospital's

rule can be used:

$$\lim_{\tilde{T}_i \to \tau_X} \frac{E_{xc}\left\{e^{X\theta^*} X_k e^{-H_0(\tilde{T}_i)e^{X\theta+C\beta}} \left(1-\tilde{T}_i/\tau_X\right)\right\}}{E_{xc}\left\{e^{X\theta^*} e^{-H_0(\tilde{T}_i)e^{X\theta+C\beta}} \left(1-\tilde{T}_i/\tau_X\right)\right\}}$$

$$= \frac{E_{xc}\left\{e^{X\theta^*} X_k e^{-H_0(\tilde{T}_i)e^{X\theta+C\beta}} /\tau_X\right\}}{E_{xc}\left\{e^{X\theta^*} e^{-H_0(\tilde{T}_i)e^{X\theta+C\beta}} /\tau_X\right\}}.$$

Other similar cases can be handled by algebraic elimination, L'Hopital's rule, or other methods to manipulate the expression.

More generally, $\frac{1}{\tilde{n}}\sum_{i=1}^{\tilde{n}} \Delta_i X_{ik}$ and $\frac{1}{\tilde{n}}\sum_{i=1}^{\tilde{n}} \Delta_i D_{ik}$ have the expectations $P(T \leq T^+)E\left(X_k|T \leq T^+\right)$ and $P(T \leq T^+)E\left(D_k|T \leq T^+\right)$ respectively, where

$$D_k = \frac{E_{xc}\left\{e^{X\theta^*} X_k e^{-H_0(T)e^{X\theta+C\beta}} S^+(T|X)\right\}}{E_{xc}\left\{e^{X\theta^*} e^{-H_0(T)e^{X\theta+C\beta}} S^+(T|X)\right\}}.$$

It can be shown using the definition of convergence in probability that $\frac{1}{\tilde{n}}\sum_{i=1}^{\tilde{n}} \Delta_i \frac{A_{\tilde{n}ik}}{B_{\tilde{n}i}}$ converges in probability to $P(T \leq T^+)E\left(D_k|T \leq T^+\right)$. Writing the conditional expectation $E\left(\cdot|T \leq T^+\right)$ as $E_{OBS}\left(\cdot\right)$ and assuming $P(T \leq T^+) \neq 0$ (i.e. the probability of observing an event is not zero), $U_{\tilde{n}k}(\theta^*)$ converges in probability to $U_k(\theta^*, \theta; \beta) = 0 = E_{OBS}(X_k - D_k)$, which leads to (4).

Web Appendix B: Proof for (7)

Using the simulation setting in Lin et al (1998) that $S^+(t|x,c) = 1 - t/\tau$ and $K = 1$, the equation (4) can be written as

$$E_{OBS}\left[\frac{E_{xc}\left\{Xe^{X\theta^*} e^{-H_0(T)e^{X\theta+C\beta}}\right\}}{E_{xc}\left\{e^{X\theta^*} e^{-H_0(T)e^{X\theta+C\beta}}\right\}}\right] = E_{OBS}(X) \qquad \text{(A-1)}$$

To be consistent with Lin et al (1998), we assume the event is sufficiently rare and $H_0(t)$ sufficiently small that $e^{H_0(t)e^{x\theta+c\beta}} \approx 1$ (i.e. $O(H_0(t)e^{x\theta+c\beta})$ can be ignored). The left side of (A-1) can then be approximated by

$$\frac{E_x\left\{Xe^{X\theta^*}\right\}}{E_x\left\{e^{X\theta^*}\right\}}.$$

The uncensoring probability can be written as

$$\begin{aligned}
\varphi_{xc} &= P(T^+ \geq T|x,c) = P(T \leq T^+|x,c) \\
&= \int P(T \leq t|x,c) P(T^+ = t)dt = \int F(t|x,c) f^+(t)dt \\
&= \int \{1 - S(t|x,c)\} f^+(t)dt = \int f^+(t)dt - \int f^+(t)e^{-H_0(t)e^{\theta x+\beta c}}dt \\
&= 1 - \int f^+(t)e^{-H_0(t)e^{\theta x+\beta c}}dt \qquad \text{(A-2)}
\end{aligned}$$

When $H_0(t)$ is small, (A-2) is approximately

$$\begin{aligned}
\varphi_{xc} &\approx 1 - \int f^+(t)\left\{1 - H_0(t)e^{\theta x+\beta c}\right\} dt \\
&= e^{\theta x+\beta c} \int f^+(t)H_0(t)dt
\end{aligned}$$

Using (6), the right side of (A-1) is therefore

$$\frac{E_{xc}(Xe^{\theta X+\beta C})}{E_{xc}(e^{\theta X+\beta C})},$$

and it follows

$$\frac{E_x\left\{e^{X\theta^*}\right\}}{E_x\left\{Xe^{X\theta^*}\right\}} = \frac{E_{xc}(e^{\theta X+\beta C})}{E_{xc}(Xe^{\theta X+\beta C})} \qquad \text{(A-3)}$$

For $X \sim B(1,P)$ and $C|x \sim B(1,\rho_x)$, (A-3) becomes

$$\begin{aligned}
1 + \frac{1-P}{P}e^{-\theta^*} &= \frac{E_x\left\{e^{\theta X}(\rho_X e^{\beta} + 1 - \rho_X)\right\}}{E_x\left\{Xe^{\theta X}(\rho_X e^{\beta} + 1 - \rho_X)\right\}} \\
&= 1 + \frac{1-P}{P}\frac{\rho_0 e^{\beta} + 1 - \rho_0}{\rho_1 e^{\beta} + 1 - \rho_1}e^{-\theta} \\
\theta^* - \theta &= \log\frac{e^{\beta}\rho_1 + (1-\rho_1)}{e^{\beta}\rho_0 + (1-\rho_0)}. \qquad \text{(A-4)}
\end{aligned}$$

For $C|x \sim N(\mu_x, \sigma^2)$

$$\begin{aligned}
1 + \frac{1-P}{P}e^{-\theta^*} &= \frac{E_x\left(e^{\theta X+\beta\mu_X+\beta^2\sigma^2/2}\right)}{E_x\left(Xe^{\theta X+\beta\mu_X+\beta^2\sigma^2/2}\right)} \\
&= 1 + \frac{1-P}{P}\frac{e^{\beta\mu_0+\beta^2\sigma^2/2}}{e^{\beta\mu_1+\beta^2\sigma^2/2}}e^{-\theta} \\
\theta^* - \theta &= \beta(\mu_1 - \mu_0). \qquad \text{(A-5)}
\end{aligned}$$

The results (A-4) and (A-5) are the same as the formulae in Lin et al (1998).

Web Appendix C: Proof for (11)

The equation (4) shows that

$$E_{OBS}\left[\frac{E_{xc}\left\{e^{X\theta^*}e^{-H_0(T)e^{X\theta+C\beta}}XS^+(T|X)\right\}}{E_{xc}\left\{e^{X\theta^*}e^{-H_0(T)e^{X\theta+C\beta}}S^+(T|X)\right\}}\right]=E_{OBS}\left[X\right] \qquad \text{(A-6)}$$

For $X \sim B(1,p)$, by the result (6), the right side of (A-6) is

$$\begin{aligned}E_{OBS}\left[X\right]&=\frac{E_{xc}(X\varphi_{XC})}{E_{xc}(\varphi_{XC})}=\frac{p\varphi_1}{p\varphi_1+(1-p)\varphi_0}\\&=p\left\{(1-p)\frac{\varphi_0}{\varphi_1}+p\right\}^{-1},\end{aligned}$$

where φ_0 and φ_1 are the uncensoring rates in control and treatment groups respectively.

The left side of (A-6) is

$$\begin{aligned}&E_{OBS}\left[\frac{E_{xc}\left\{e^{X\theta^*}e^{-H_0(T)e^{X\theta+C\beta}}XS^+(T|X)\right\}}{E_{xc}\left\{e^{X\theta^*}e^{-H_0(T)e^{X\theta+C\beta}}S^+(T|X)\right\}}\right]\\
=&E_{OBS}\left[\frac{pS^+(T|x=1)E_{c|x=1}\left\{e^{\theta^*}e^{-H_0(T)e^{\theta+C\beta}}\right\}}{pS^+(T|x=1)E_{c|x=1}\left\{e^{\theta^*}e^{-H_0(T)e^{\theta+C\beta}}\right\}+(1-p)S^+(T|x=0)E_{c|x=0}\left\{e^{-H_0(T)e^{C\beta}}\right\}}\right]\\
=&pE_{OBS}\left[\left\{p+(1-p)e^{-\theta^*}\frac{S^+(T|x=0)E_{c|x=0}\left(e^{-H_0(T)e^{\beta C}}\right)}{S^+(T|x=1)E_{c|x=1}\left(e^{-H_0(T)e^{\theta+\beta C}}\right)}\right\}^{-1}\right].\end{aligned}$$

These lead to (9).

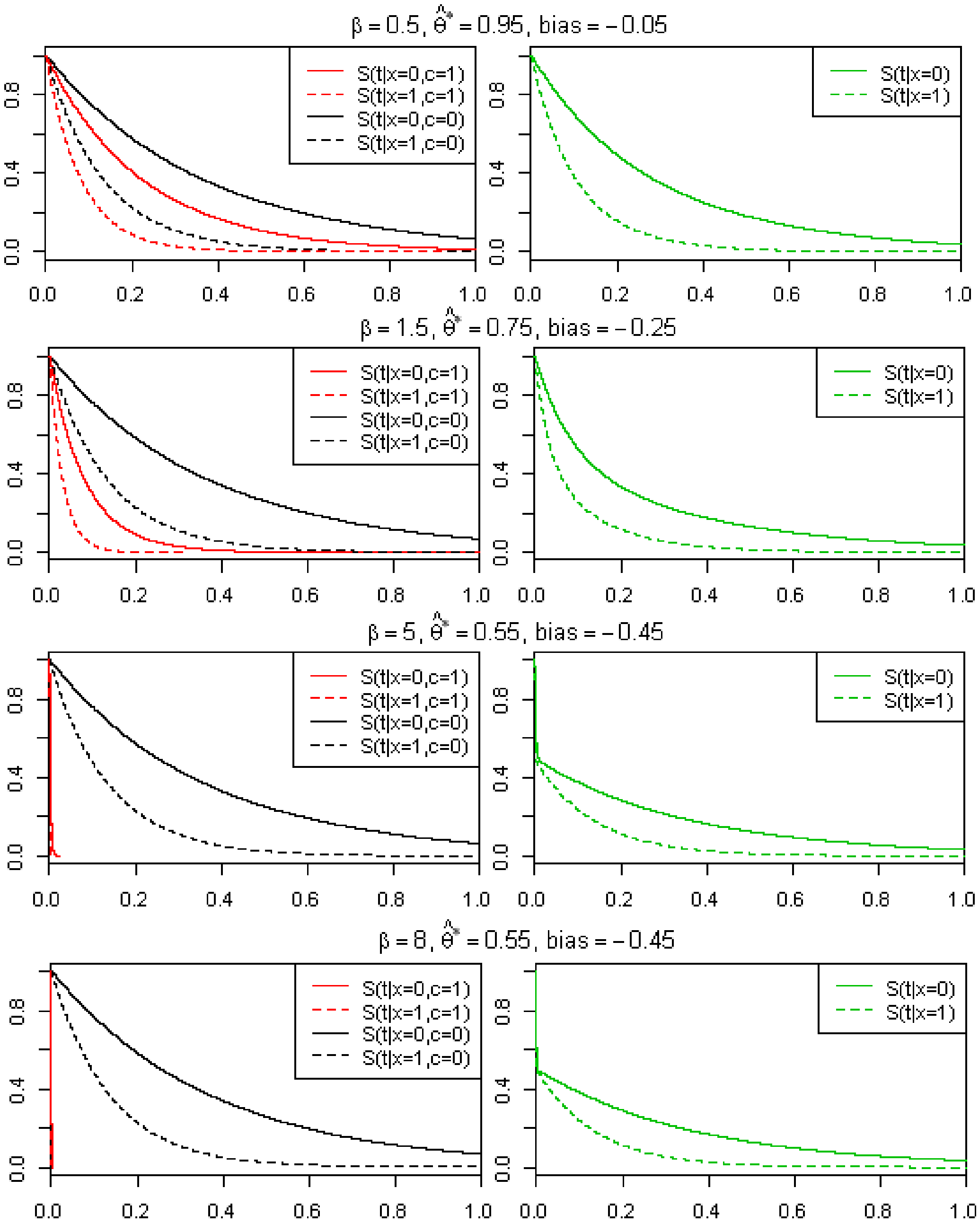

Web figure 1: Kaplan-Meier plots for complete data $S(t|x,c)$ (left) and the observed data $S(t|x)$ (right). The sample size is 10,000. The data were generated from $h(t|x,c) = e^{x+\beta c}, X \sim B(1, 0.5)$ and $C \sim B(1, 0.5)$. Under this setting, $S(t|x) = 0.5S(t|x, c = 1) + 0.5S(t|x, c = 0)$. As β increases from 0.5 to 5, on the left side, $S(t|x, c = 1)$ tend to zero, reducing the observed difference between $S(t|x = 1)$ and $S(t|x = 0)$ (right). As a consequence, the marginal hazard ratio $\exp(\hat{\theta}^*) = h(t|x = 1)/h(t|x = 0)$, attenuates to 1 and the bias increases from -0.14 to -0.45. But as β increases from 5 to 8, there is no apparent change in the difference $S(t|x = 1)$ and $S(t|x = 0)$. As a result, the bias does not increase with β and -0.45 is the limit of bias.

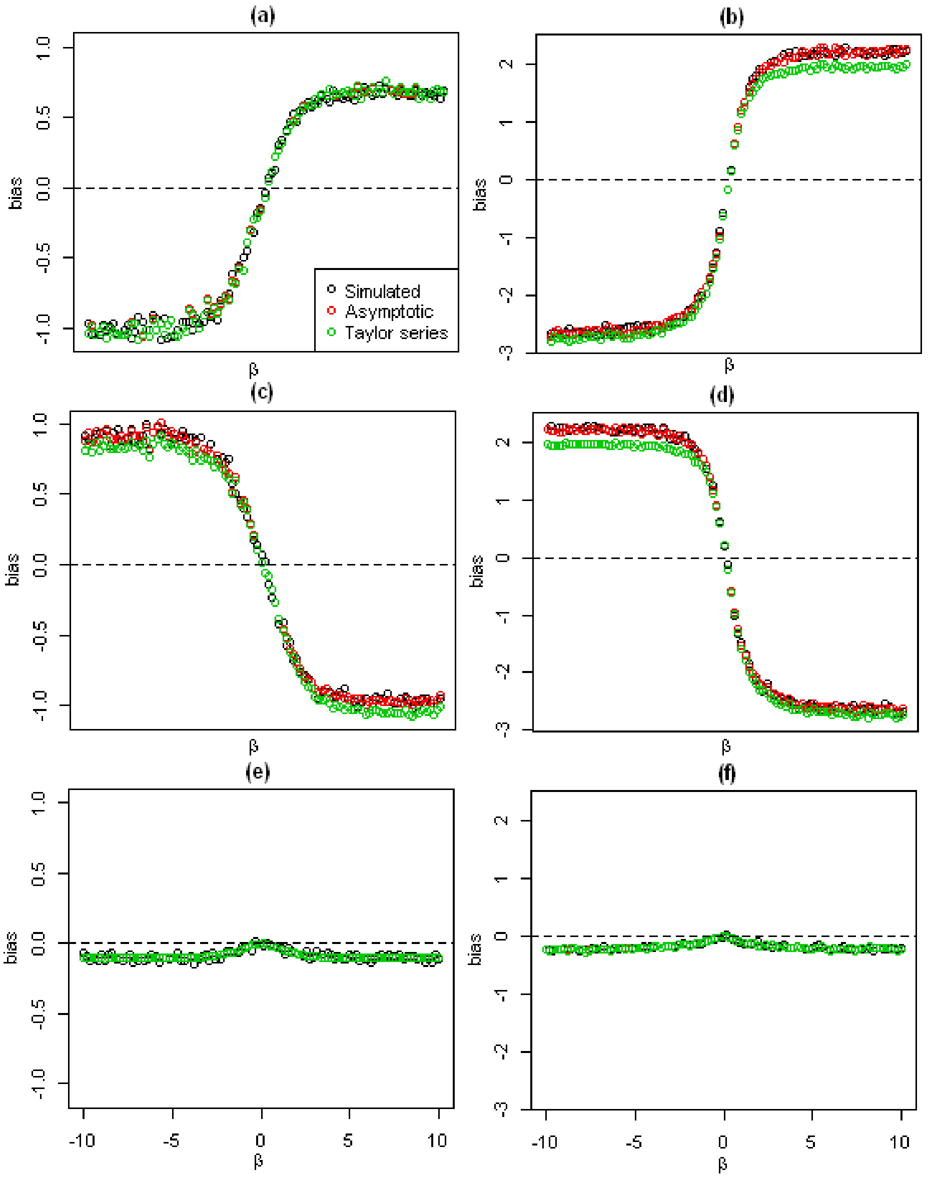

Web figure 2: Comparison of simulated biases, asymptotic biases and first-order Taylor series approximations for different types of omitted covariate and censorship when $\theta = 0.25$: (a) Binary confounder C: $(\rho_0 = 0.3, \rho_1 = 0.7)$, censored; (b) Normal confounder C: $(\mu_0 = -1, \mu_1 = 1)$, censored; (c) Binary confounder C: $(\rho_0 = 0.7, \rho_1 = 0.3)$, censored; (d) Normal confounder C: $(\mu_0 = 1, \mu_1 = -1)$, censored; (e) Binary balanced C: $(\rho_0 = \rho_1 = 0.5)$, uncensored; (f) Normal balanced C: $(\mu_0 = \mu_1 = 0)$, uncensored.

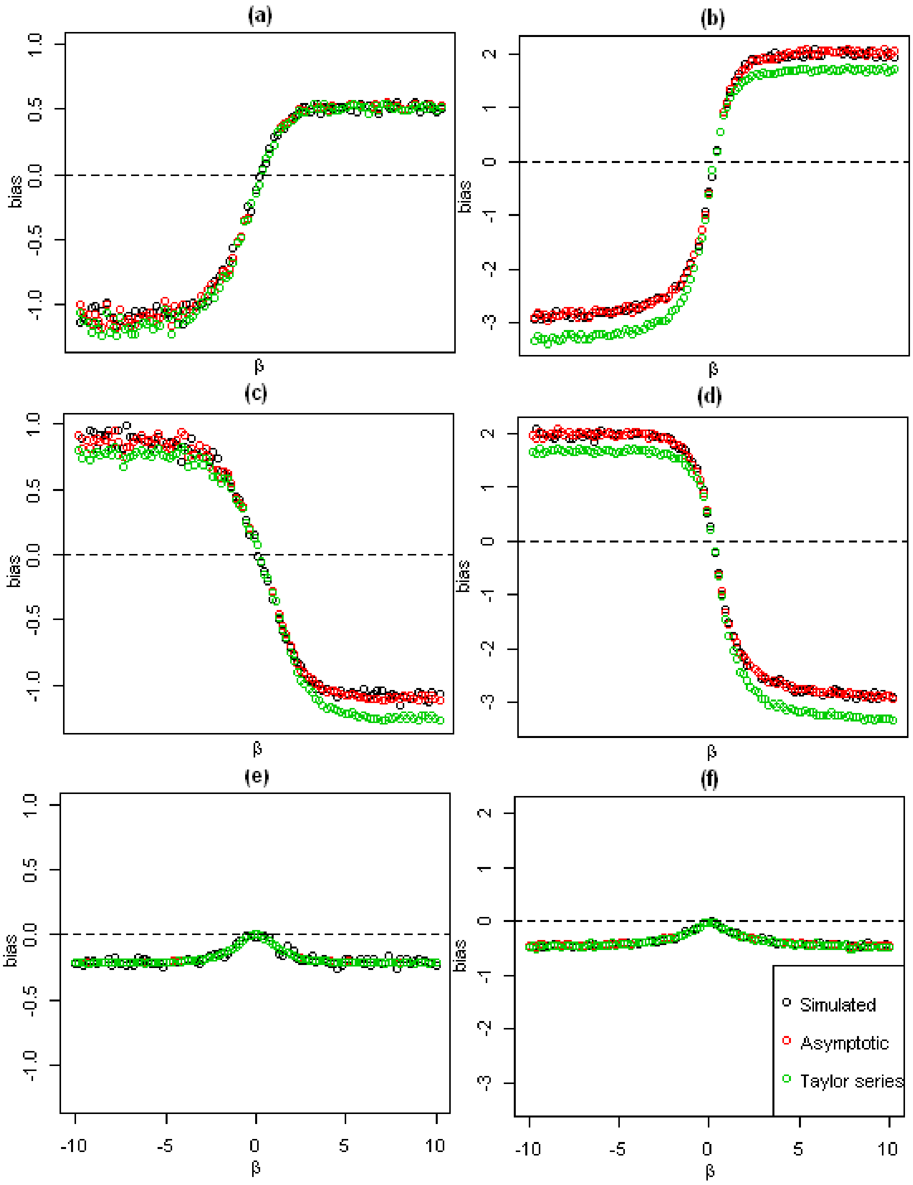

Web figure 3: Comparison of simulated biases, asymptotic biases and first-order Taylor series approximations for different types of omitted covariate and censorship when $\theta = 0.5$: (a) Binary confounder C: $(\rho_0 = 0.3, \rho_1 = 0.7)$, censored; (b) Normal confounder C: $(\mu_0 = -1, \mu_1 = 1)$, censored; (c) Binary confounder C: $(\rho_0 = 0.7, \rho_1 = 0.3)$, censored; (d) Normal confounder C: $(\mu_0 = 1, \mu_1 = -1)$, censored; (e) Binary balanced C: $(\rho_0 = \rho_1 = 0.5)$, uncensored; (f) Normal balanced C: $(\mu_0 = \mu_1 = 0)$, uncensored.

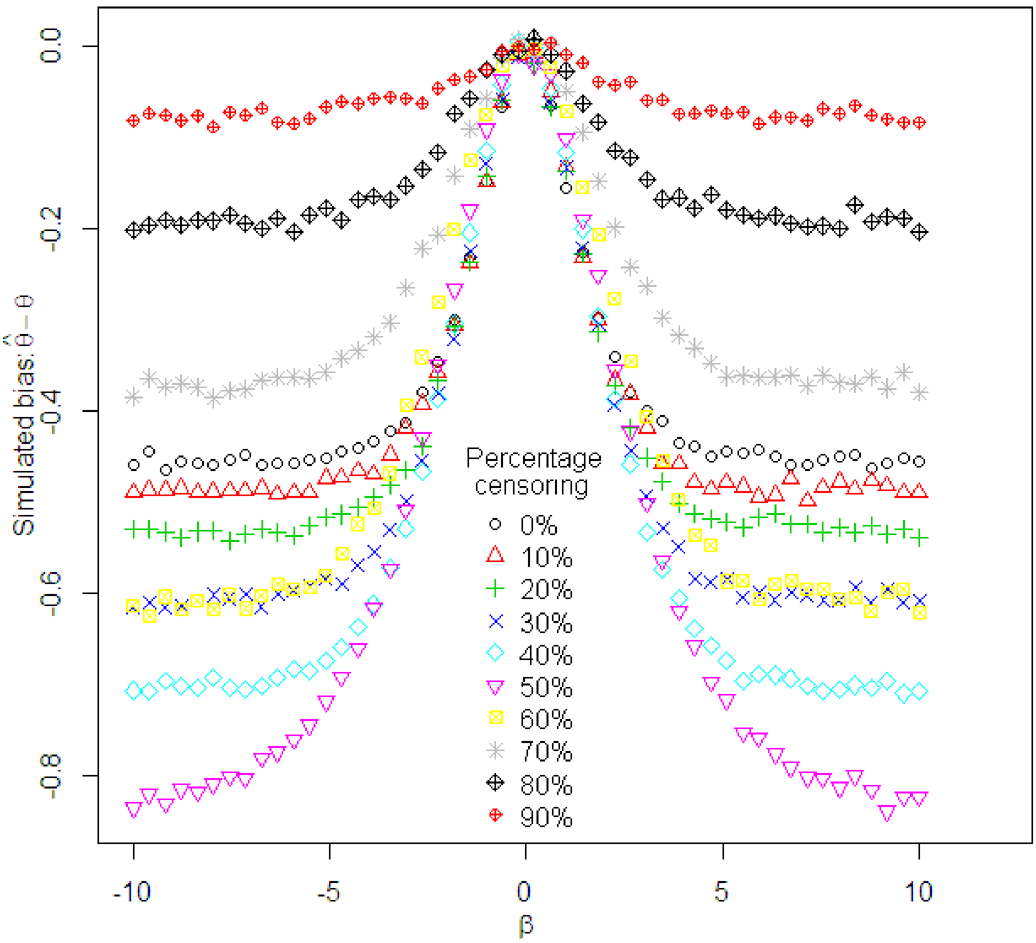

Web figure 4: Bias of omitting a balanced covariate under different censoring percentages. In this simulation, γ_0 and γ_1 are solved from $1 - \varphi = E_{c|x}\left\{\frac{1-e^{-e^{\gamma_0+(\gamma_1+\theta)x+\beta c}}}{e^{\gamma_0+(\gamma_1+\theta)x+\beta c}}\right\}$ for $x = 0$ and 1. It can be seen that γ_1 should be $-\theta$ to ensure that the censoring probabilities are the same in the two treatment groups. The overall censoring probability is controlled by the value of γ_0.

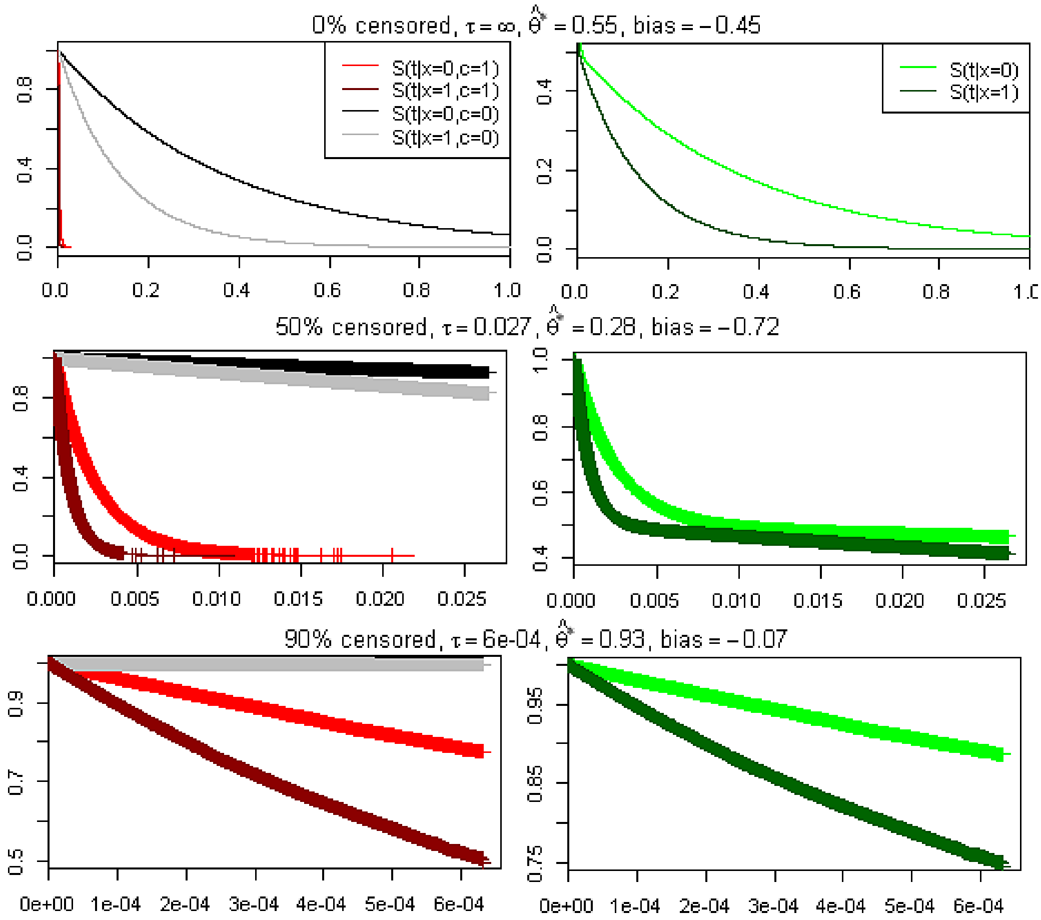

Web figure 5: Kaplan-Meier plots for complete data $S(t|x,c)$ (left) and the observed data $S(t|x)$ (right). The sample size is 200,000. The data were generated from $h(t|x,c) = e^{x+5c}$, $X \sim B(1,0.5)$ and $C \sim B(1,0.5)$. $S(t|x) = 0.5S(t|x,c=1) + 0.5S(t|x,c=0)$. For ease of simulation, we generated t^+ from uniform $(0,\tau)$, where τ was solved from (13) such that the overall proportions censored could be 0%, 50% or 90%. When 0% data are censored, $S(t|x,c=1)$ gets close to zero for $t < 0.05$ and so most of the information about θ (i.e. the difference between $S(t|x=1)$ and $S(t|x=0)$) is supplied by the difference between $S(t|x=1,c=0)$ and $S(t|x=0,c=0)$. When 50% data are censored, the times with $c=0$ are more likely to be censored than those with $c=1$. As a consequence, the difference between $S(t|x=1,c=0)$ and $S(t|x=0,c=0)$ cannot supply much information about θ and so the bias increases. As the censoring percentage increases from 50% to 90%, the difference between $S(t|x=1,c=0)$ and $S(t|x=0,c=0)$ becomes less informative. At the same time, the difference between $S(t|x=1,c=1)$ and $S(t|x=0,c=1)$ starts to supply more of the information about θ and the bias decreases. When 90% of the data are censored, almost all of the few deaths observed will occur in subjects with the same value of c ($c=1$) and the bias is very low.

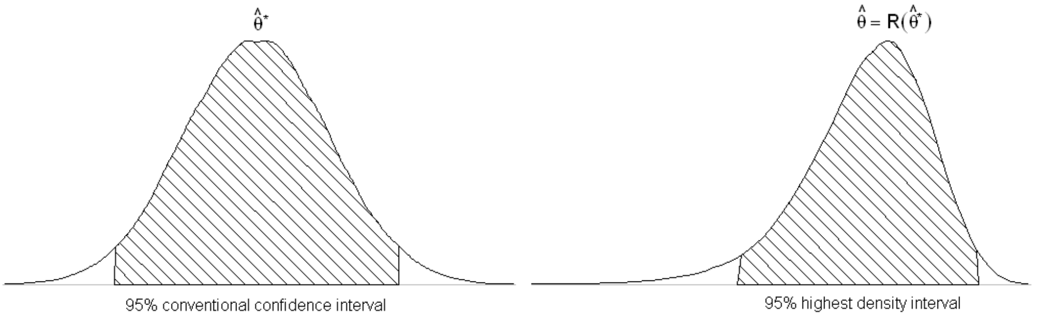

Web figure 6: Illustrative example showing density plots of 100,000 bootstrap samples of $\hat{\theta}^*$ and the corresponding $\hat{\theta} = R(\hat{\theta}^*)$. A sample of size 200 was generated from $h(t|x,c) = e^{x+3c}, X \sim B(1,0.5), C|x \sim B(1,0.3+0.4x)$ and $T^+ \sim uniform(0,1)$. In this simulation, the distribution of $\hat{\theta} = R(\hat{\theta}^*)$ is slightly right skewed. It will be not accurate to use the standard error of the bootstrap sample of $\hat{\theta} = R(\hat{\theta}^*)$ to construct the C.I.

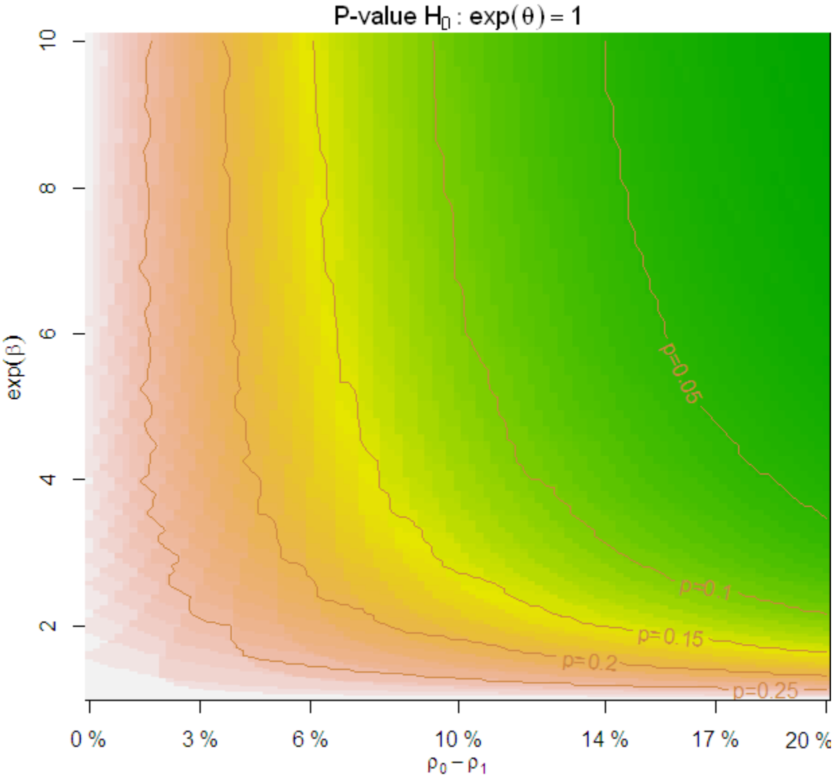

Web figure 7: Sensitivity analysis for the p-value for the treatment effect of folinic acid on age of sitting for children with Down's syndrome.

Web table 1: Simulated bias of point estimates and coverage of 95% confidence intervals for the hazard ratio associated with treatment under two methods of sensitivity analysis, when censoring is light

					Fraction	unadjusted		Lin et al (1998)		$\hat{\theta}=R(\hat{\theta}^*)$	
$\widetilde{n}$	τ	β	ρ_0	ρ_1	Censored	Bias	Coverage	Bias	Coverage	Bias	Coverage
100	5.40	1	0.1	0.9	10%	0.74	22%	-0.03	95%	0.05	78%
	5.06		0.3	0.7	10%	0.27	79%	-0.11	82%	0.05	77%
	4.65		0.5	0.5	10%	-0.14	89%	-0.14	89%	-0.01	88%
	4.94	2	0.1	0.9	10%	1.05	3%	-0.37	65%	0.05	82%
	4.47		0.3	0.7	10%	0.26	81%	-0.37	63%	0.00	82%
	3.80		0.5	0.5	10%	-0.31	67%	-0.31	67%	0.01	92%
	4.69	3	0.1	0.9	10%	1.16	2%	-0.67	34%	0.12	69%
	4.14		0.3	0.7	10%	0.17	89%	-0.59	29%	-0.08	89%
	3.52		0.5	0.5	10%	-0.41	56%	-0.41	56%	0.00	91%
500	5.47	1	0.1	0.9	10%	0.70	0%	-0.08	81%	0.02	76%
	5.07		0.3	0.7	10%	0.22	45%	-0.15	67%	0.01	84%
	4.63		0.5	0.5	10%	-0.14	73%	-0.15	73%	0.00	91%
	4.94	2	0.1	0.9	10%	0.96	0%	-0.45	11%	-0.01	69%
	4.37		0.3	0.7	10%	0.24	36%	-0.39	4%	0.03	90%
	3.82		0.5	0.5	10%	-0.32	9%	-0.32	9%	-0.01	89%
	4.77	3	0.1	0.9	10%	1.11	0%	-0.72	2%	0.04	74%
	4.11		0.3	0.7	10%	0.19	57%	-0.57	1%	-0.02	90%
	3.55		0.5	0.5	10%	-0.41	1%	-0.41	1%	0.02	91%
1000	5.46	1	0.1	0.9	10%	0.67	0%	-0.10	74%	0.00	75%
	5.06		0.3	0.7	10%	0.22	17%	-0.16	47%	0.01	82%
	4.66		0.5	0.5	10%	-0.14	47%	-0.14	47%	0.01	86%
	4.94	2	0.1	0.9	10%	0.98	0%	-0.44	1%	0.01	68%
	4.39		0.3	0.7	10%	0.22	16%	-0.41	0%	0.00	85%
	3.82		0.5	0.5	10%	-0.33	0%	-0.33	0%	-0.01	92%
	4.72	3	0.1	0.9	10%	1.09	0%	-0.74	0%	0.03	70%
	4.15		0.3	0.7	10%	0.20	19%	-0.56	0%	0.00	90%
	3.53		0.5	0.5	10%	-0.42	0%	-0.42	0%	0.00	89%

Web table 2: Simulated bias of point estimates and coverage of 95% confidence intervals for the hazard ratio associated with treatment under two methods of sensitivity analysis, when censoring is heavy

					Fraction	unadjusted		Lin et al (1998)		$\hat{\theta}=R(\hat{\theta}^*)$	
$\widetilde{n}$	τ	β	ρ_0	ρ_1	Censored	Bias	Coverage	Bias	Coverage	Bias	Coverage
100	0.06	1	0.1	0.9	90%	4.52	99%	3.73	97%	2.28	100%
	0.06		0.3	0.7	90%	3.50	100%	3.12	95%	0.12	100%
	0.06		0.5	0.5	90%	0.92	99%	0.92	99%	-0.81	100%
	0.02	2	0.1	0.9	90%	10.77	99%	9.36	99%	6.01	100%
	0.03		0.3	0.7	90%	4.19	100%	3.56	98%	1.80	100%
	0.03		0.5	0.5	90%	0.41	96%	0.41	96%	-1.36	100%
	0.01	3	0.1	0.9	90%	10.74	99%	8.91	96%	8.57	100%
	0.01		0.3	0.7	90%	4.84	100%	4.08	97%	2.19	100%
	0.01		0.5	0.5	90%	0.36	98%	0.36	98%	-1.27	100%
500	0.06	1	0.1	0.9	90%	0.81	49%	0.03	95%	-0.35	100%
	0.06		0.3	0.7	90%	0.42	84%	0.05	94%	-0.39	100%
	0.06		0.5	0.5	90%	0.02	97%	0.02	97%	-0.30	100%
	0.02	2	0.1	0.9	90%	2.04	9%	0.63	97%	-0.38	97%
	0.03		0.3	0.7	90%	0.71	56%	0.08	100%	-0.18	100%
	0.03		0.5	0.5	90%	-0.02	94%	-0.02	94%	-0.15	100%
	0.01	3	0.1	0.9	90%	2.16	2%	0.33	98%	-0.07	99%
	0.01		0.3	0.7	90%	0.82	47%	0.06	97%	-0.12	100%
	0.01		0.5	0.5	90%	-0.07	94%	-0.07	94%	-0.29	100%
1000	0.06	1	0.1	0.9	90%	0.80	17%	0.02	94%	-0.07	100%
	0.06		0.3	0.7	90%	0.34	77%	-0.04	92%	-0.02	100%
	0.06		0.5	0.5	90%	-0.03	98%	-0.03	98%	-0.14	100%
	0.02	2	0.1	0.9	90%	1.48	0%	0.07	95%	-0.02	100%
	0.03		0.3	0.7	90%	0.64	30%	0.01	97%	-0.02	100%
	0.03		0.5	0.5	90%	-0.04	97%	-0.04	97%	-0.08	100%
	0.01	3	0.1	0.9	90%	1.97	0%	0.14	96%	0.05	100%
	0.01		0.3	0.7	90%	0.76	16%	0.00	95%	-0.04	100%
	0.01		0.5	0.5	90%	-0.06	97%	-0.06	97%	-0.21	100%

Web table 3: Point estimates and 95% confidence intervals for the hazard ratio for age at sitting associated with antioxidant supplementation after adjustment for an unmeasured binary confounder of specified properties. The unadjusted estimate and 95% confidence interval are 1.10 (0.77, 1.56).

		ρ_0				
exp(β)	ρ_1	0.1	0.3	0.5	0.7	0.9
2	0.1	1.17<br>(0.70, 1.66)	1.33<br>(0.79,1.89)			
	0.3		1.18<br>(0.69,1.69)	1.36<br>(0.79,1.94)		
	0.5			1.19<br>(0.69,1.71)	1.37<br>(0.79,1.98)	
	0.7				1.18<br>(0.69,1.71)	1.37<br>(0.80,1.98)
	0.9					1.18<br>(0.70,1.67)
6	0.1	1.19<br>(0.68, 1.70)	1.54<br>(0.89, 2.25)			
	0.3		1.22<br>(0.64,1.85)	1.70<br>(0.89,2.63)		
	0.5			1.25<br>(0.61,2.02)	1.87<br>0.92,3.03)	
	0.7				1.25<br>(0.62,2.08)	1.88<br>(0.94,3.13)
	0.9					1.21<br>(0.67,1.85)
10	0.1	1.19<br>(0.67, 1.71)	1.60<br>(0.91, 2.36)			
	0.3		1.23<br>(0.61,1.91)	1.83<br>(0.93,2.93)		
	0.5			1.28<br>(0.57,2.15)	2.12<br>0.96,3.69)	
	0.7				1.29<br>(0.58,2.28)	2.18<br>(**1**,3.98)
	0.9					1.22<br>(066,1.95)

Web table 4: Simulation study based on a real example of a randomised controlled trial with size $\tilde{n} = 140$ and light censorship. The number of replications is 1000. Point estimates and coverage of confidence intervals are shown using the proposed method of sensitivity analysis (equations (14) and (16)). Survival times were simulated assuming a true treatment effect of $\exp(\theta) = 1.5$ and a binary omitted covariate with $\exp(\beta) = 6$ (for three different levels of confounding, $\rho_0 - \rho_1$). The results show that, under conditions similar to those in the trial, the proposed method of sensitivity analysis can give unbiased point estimates with good coverage of C.I. for clinically relevant treatment effects in the presence of substantial unmeasured confounding.

					Fraction	Mean(Coverage)		
$\widetilde{n}$	τ	$\exp(\theta)$	$\exp(\beta)$	(ρ_0, ρ_1)	Censored	unadjusted	Lin et al (1998)	$e^{R(\hat{\theta}^*)}$
140	3	1.5	6	(0.3,0.1)	22%	1.16(71%)	1.93(77%)	1.55(97%)
				(0.5,0.3)	18%	1.08(54%)	1.51(95%)	1.53(97%)
				(0.7,0.5)	13%	1.01(40%)	1.30(88%)	1.50(97%)
				(0.9,0.7)	9%	0.98(31%)	1.19(71%)	1.51(98%)

Web table 5: Simulation study based on a real example of a non-randomised study with size $\tilde{n} = 1043$ and light censorship (15.7%). Here the treatment variable is a continuous exposure, and so equation (8) was used to assess the performance of the method of Lin et al (1998). The number of replications is 1000. The false rejection rate is the probability that the valid null hypotheses $H_0 : \theta = 0$ is incorrectly rejected. As the true value is $\theta = 0$, the confounding bias dominates the total missing covariate bias. The extended method of Lin et al (1998) thus can give unbiased point estimates in this simulation. However the proposed method of sensitivity analysis has a much lower false rejection rate than the extended method of Lin et al (1998) as the correlation between the exposure and unmeasured confounder becomes large.

			Fraction		Mean(False Rejection Rate)		
$\widetilde{n}$	$\exp(\theta)$	$\exp(\beta)$	Censored	$corr(X, C)$	Unadjusted	Equation (7)	$e^{R(\hat{\theta}^*)}$
1043	1	6	15.7%	0.3	1.06(100%)	1.00 (6%)	1.00 (2%)
				0.5	1.10(100%)	1.01 (8%)	1.00 (3%)
				0.7	1.15(100%)	1.02 (47%)	1.01 (10%)

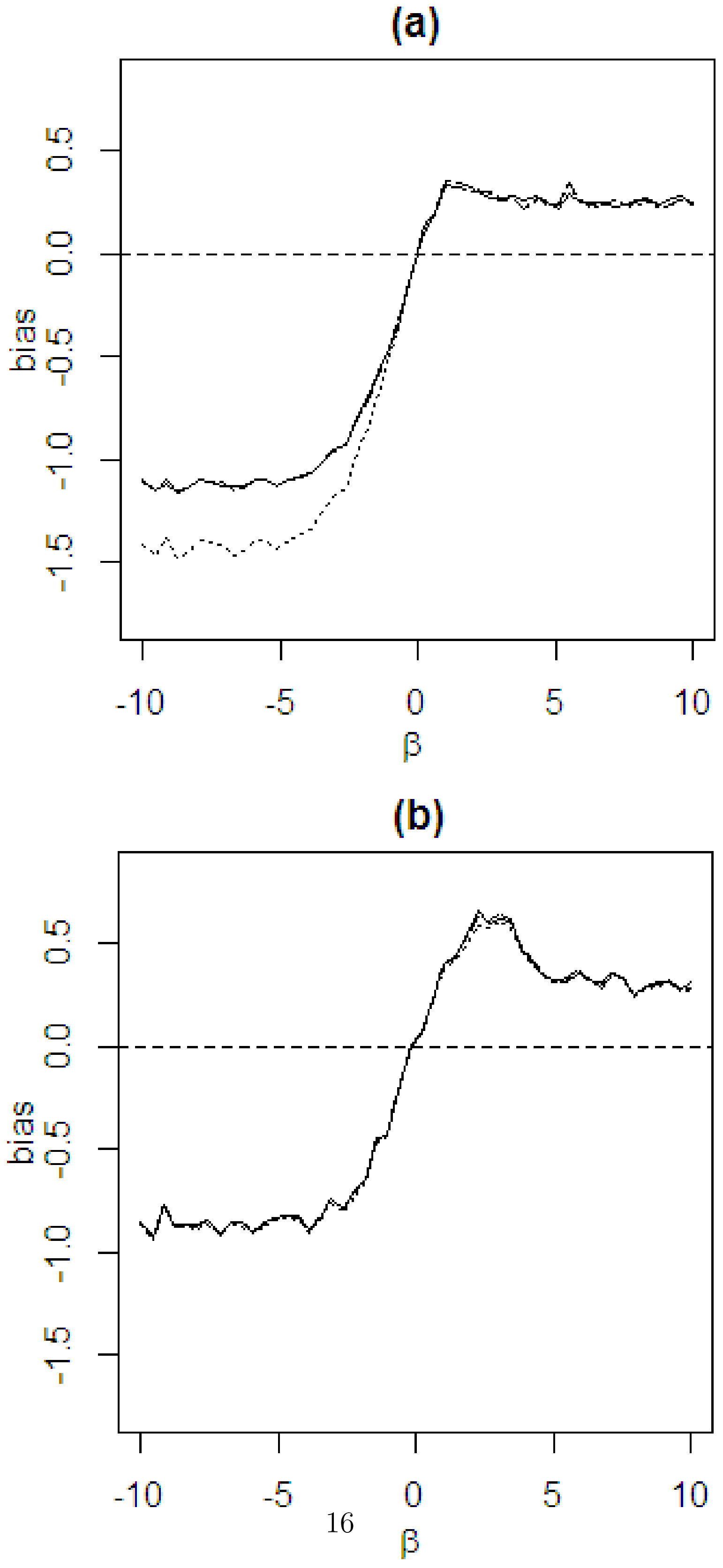

Web figure 8: Bias plots for the scenario in Fig 1 (a) but with different censoring distributions: (a) $T^{+} \sim uniform(0, 0.5e^{x})$ and (b) $T^{+} \sim uniform(0, e^{-3x})$. The plots are slightly different from the original plot with $\tau = 1$, although the general patterns are broadly similar.

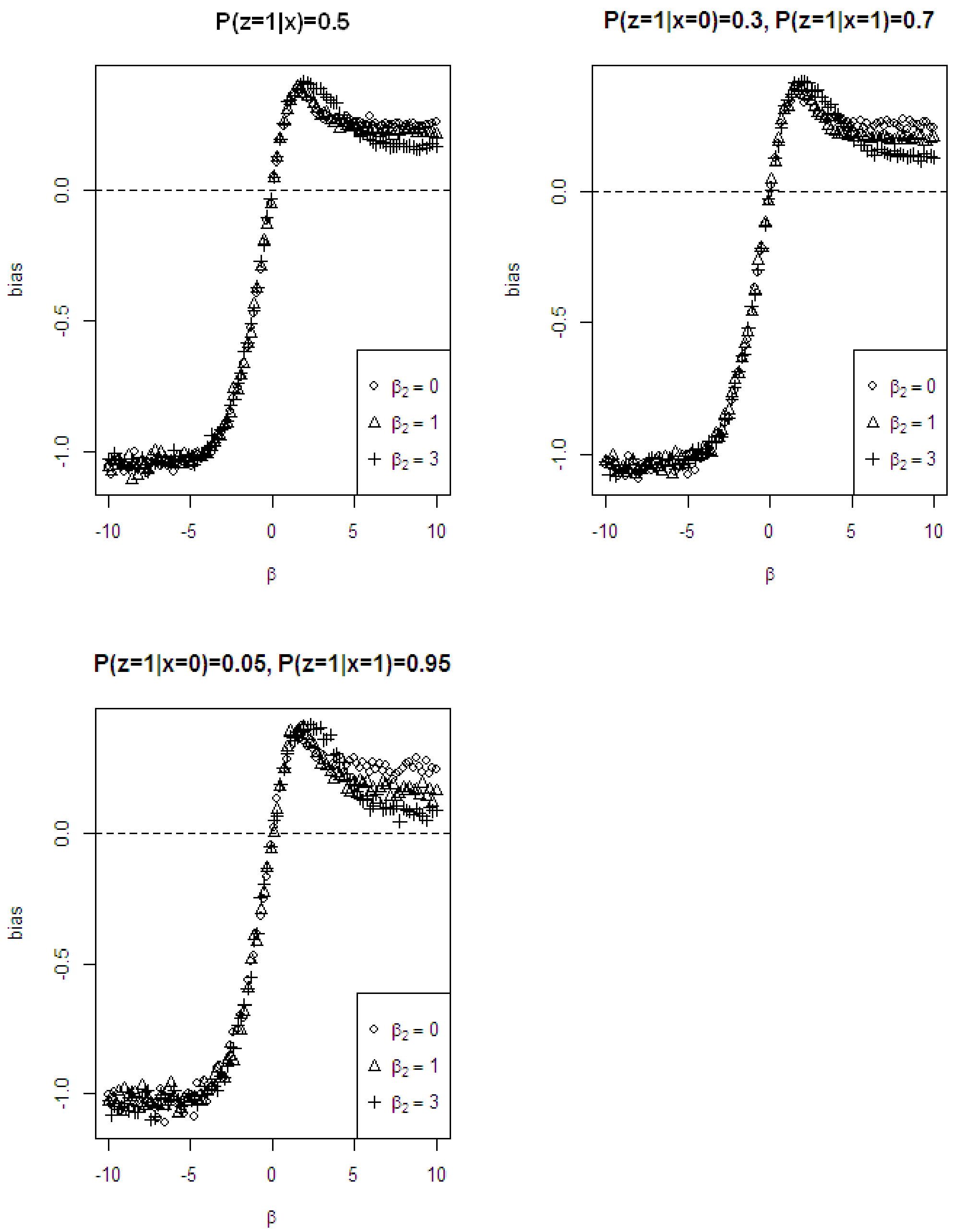

Web figure 9: The effect of additional measured covariates on the simulated bias when $T^+ \sim Uniform(0, e^{-\theta x - \beta_2 z})$. The plots show some differences from the original plots with $T^+ \sim Uniform(0, 1)$ but again the bias changes little when the additional covariate Z is added to the model.
